# Motivational factors of nurses in a group of primary health centres in the city of Lisbon: qualitative study

**DOI:** 10.1080/07853890.2021.1895984

**Published:** 2021-09-28

**Authors:** Claudina Martins, Teresa Potra, Francisca Lucas, Pedro Bernardes Lucas

**Affiliations:** a Agrupamento de Centros de Saúde Lisboa Central, Lisboa, Portugal; b Unidade de Investigação e Desenvolvimento em Enfermagem da Escola Superior de Enfermagem de Lisboa, Lisboa, Portugal; c Escola Superior de Enfermagem de Lisboa, Lisboa, Portugal

## Abstract

**Introduction:**

Professionals motivation, as a determinant of the behaviour in organisations, is an important factor for their efficiency. Managing people while keeping them motivated to work is not an easy task and because of that it is considered one of the most difficult and complex functions of the manager [1]. Because of that, the knowledge based on causes that move or influence nurses’ motivation is considered important as a management instrument [2]. Sensitivities to this topic and considering the current conjuncture, in which Portuguese nurses have been confronted with low salaries [3] and work overload due to the low number of nurses per 1000 inhabitants [4], the starting point of this study is about the question: which factors can motivate nurses from a Lisbon primary care units? The aim is meeting the motivational factors of nurses from a Lisbon primary care unit. Specific aims are: understanding which factors motivate nurses from primary care unit in Lisbon, understanding motivational influence due to nursing practice and nursing management.

**Materials and methods:**

Qualitative case study, exploratory and descriptive approach. The data collection was based on a semi-structured interview based on a predefined script performed with 9 nurses from a Lisbon primary care unit, from 18 January to 5 February 2019, who agreed to participate in the study. The number of participants was determined following the response saturation criteria [5]. In order to increase data variety, interviews were conducted with nurses working in different types of Functional Units. The age of the subjects ranged between 33 and 46 years old, with an average professional practice time of 17 years. Interviews were recorded, fully transcribed and analysed according to Bardin [6] content analysis. The study started after obtaining approval from the Executive Director of the Group of Primary Care Units, and the Health Ethics Committee of the respective Regional Health Administration and informed consent signed by the participants.

**Results:**

Study results indicate that the main motivational factor of nurses is: rewards, career development, recognition, workplace/practice environment, financing based on performance, leadership and professional achievement. Career development was the factor in which participants attributed greater importance and on the other hand, financing based on performance was considered the less important as a motivational factor.

**Discussion and conclusions:**

Despite of participants’ motivational factors being identical, it was verified that their importance and meaning are different to which one of them. According that, context indicators emerged with contradictory meaning. This enhances the individuality of each participant while being unique and motivated by their own needs. It was noticed that motivation is not maximum on most of the participants and that there is also dissatisfaction with the measures that could work as motivators. Managers should take into account scientific evidence concerning nurses’ motivational factors in order to improve their motivation. Further studies should be developed, particularly in primary health care, where the lack of scientific content is notorious.

## References

[1] Cunha MP, Rego A, Cunha RC, et al. Manual de comportamento organizacional e gestão., 8th ed.; Lisboa (Portugal): Editora RH; 2016.

[2] Andrade CVS. Motivação no trabalho e perceção da orientação empreendedora dos enfermeiros. Dissertação de mestrado. Lisboa (Portugal): Escola Superior de Enfermagem de Lisboa; 2013.

[3] OECD/European Observatory on Health Systems and Policies. Portugal: Perfil de Saúde do País 2019. OECD Publishing; 2019. Available from:

[4] OECD/European Observatory on Health Systems and Policies. Portugal: Perfil de Saúde do País 2017. OECD Publishing; 2017. Available from: 10.1787/9789264285385-pt

[5] Polit DF, Beck CT, Hungler BP. Fundamentos de Pesquisa em Enfermagem – Métodos, avaliação e utilização. 5th ed. Porto Alegre (Portugal): Artemed; 2004.

[6] Bardin L. Análise de Conteúdo. São Paulo (Brasil): Edições 70; 2016.

